# Sialometry of Upper Labial Minor Glands: A Clinical Approach by the Use of Weighing Method Schirmer's Test Strips Paper

**DOI:** 10.1155/2014/268634

**Published:** 2014-03-09

**Authors:** Denise Pinheiro Falcão, Soraya Coelho Leal, Celi Novaes Vieira, Andy Wolff, Tayana Filgueira Galdino Almeida, Fernanda de Paula e Silva Nunes, Rivadávio Fernandes Batista de Amorim, Ana Cristina Bezerra

**Affiliations:** ^1^Department of Pathology, Faculty of Medicine, University of Brasilia, Campus Universitario Darcy Ribeiro, 70910-900 Brasilia, DF, Brazil; ^2^Department of Dentistry, Faculty of Health Science, University of Brasilia, Campus Universitario Darcy Ribeiro, 70910-900 Brasilia, DF, Brazil; ^3^Private Practice, 65 Hatamar Street Harutzim, 60917 Harutzim, Israel; ^4^Private Practice, SCN Qd.5 No. 50 torre norte sala 1122, 70715-900 Brasilia, DF, Brazil

## Abstract

*Objectives*. To establish referential values ranges of hyposalivation and normosalivation for the salivary flow rate (SFR) of upper labial (LS) and palatal (PS) mucosa using Schirmer's test strips paper and as a second goal to determine the values ranges of the SFR of palatal (PS) and upper labial (LS) mucosa in subjects with and without xerostomia. *Methods*. A cross-sectional study was conducted among subjects distributed in three groups according to their unstimulated and stimulated whole saliva. *Results*. 144 subjects were enrolled in groups as follows: severe hyposalivation (*n* = 24), mild hyposalivation (*n* = 78), and normosalivation (*n* = 42). The mean and the 95% confidence interval for the LS flow rate (**μ**L/cm^2^/min) were 3.2 (2.46 to 3.94), 5.86 (4.96 to 6.75), and 9.08 (7.63 to 10.53) (*P* < 0.001) for each group, respectively. The PS results were 1.01 (0.68 to 1.34), 1.72 (1.31 to 2.13), and 2.44 (1.66 to 3.22) (*P* = 0.014). Xerostomia complainers presented lower rates of LS (5.17 (4.06 to 6.23)) than non-complainers (7.33 (6.4 to 8.27)) (*P* = 0.003). *Conclusions*. The test was reliable to provide referential values ranges for LS flow rate measurement and was shown to be valid to distinguish normosalivation from severe and mild hyposalivation and also to predict xerostomia.

## 1. Introduction

The major salivary glands along with 300–500 minor salivary glands produce about 0.5 to 0.6 liters of whole saliva daily [1]. The unstimulated flow rate usually accounts for 0.3 to 0.4 milliliters per minute (mL/min), but the range is wide [2]. Even though minor glands secrete only 6–10% of whole saliva [3], they have strategic functions on oral protection due to their secreted blood-group substances, like IgA, antigens ABH, Lewis, and others [4–6]. Furthermore, they play a role in the perception of dry mouth following changes in their saliva composition [7–9]. Decreased labial gland salivation has been reported to affect subjective feelings of dry mouth both in individuals with normal and subnormal whole saliva flow [10].

Some methods have been proposed for sialometry of unstimulated and stimulated whole saliva (UWS and SWS) from major glands [11–13], but fewer studies have specifically investigated minor salivary glands flow rate. A possible explanation for this can be partially attributed to the difficulties experienced in collecting and quantifying saliva from minor glands [4, 14].

Schirmer's test is used routinely by ophthalmologists to measure tear film wetness as one of the objective ocular components of the American European classification criteria for identifying Sjögren Syndrome (SS) [15]. As filter paper strips have been previously used for sialometry [16–19], the aim of this study was to establish referential values range of hyposalivation and normosalivation for the salivary flow rates of soft palate (PS) and upper labial (LS) mucosa areas using a weighing method with Schirmer's test strips paper and as a second goal to verify their range values in subjects with and without xerostomia.

## 2. Materials and Methods

### 2.1. Sample

The study population consisted of patients attending the dentistry clinic of the Hospital of the University of Brasilia, Brazil, over a six-month period. Subjects were enrolled in a consecutive manner and informed consent was obtained from each one. The human subject protocol was in accordance with the principles laid down in the Declaration of Helsinki and was approved by the Ethics Committee of the Medicine Faculty of University of Brasília (0055/2009).

The sample included smokers, nonsmokers, healthy individuals, and also individuals with mild chronic disease, for example, hypertension, diabetes, depression, and SS and endocrinological diseases like hyperthyroidism and hypothyroidism, either under pharmacological treatment or not.

Saliva collections were performed between 8:00 and 10:00 a.m. Subjects were instructed not to smoke, eat, drink, or perform any oral hygiene procedures for at least 2 hours prior to the measurements. Nevertheless, the individuals were instructed to drink 300 mL of water 2 hours preceding saliva collections to avoid that the variability in the hydration of the body could affect the results. Before the saliva collections, subjective oral dryness was assessed by the following question: “*How often do you feel your mouth dry?*” Individuals answering “always,” “almost always,” and “frequently” were classified as having xerostomia [24].

The weighing method was performed to assess salivary flow and an electronic balance (AW 320, Shimadzu Corporation, Kyoto, Japan) was used for this purpose. Considering that each 1 gram corresponds to 1 mL of saliva (10), the difference in weight of the vial collection, before and after sampling, divided by the period time used, gave the values. The samples of saliva of each participant were performed in a single session. The sequence was firstly from the soft palate area, followed by the upper labial mucosa, UWS, and SWS. Minor glands saliva was firstly collected to avoid possible additional stimulus from the whole saliva collection.

Subjects were distributed among three groups based on their UWS and also SWS [25] as follows: severe hyposalivation group = UWS flow rate <0.1 mL/min and SWS flow rate <0.7 mL/min; normosalivation group = UWS flow rate >0.3 and SWS flow rate ≥1.0 mL/min [25]; mild hyposalivation group = individuals with UWS 0.1 to 0.3 and SWS 0.7 to 1.0 mL/min, or only either of UWS or SWS with normal values.

### 2.2. Collection of Saliva from Minor Salivary Glands

Cotton rolls were placed on Stensen's duct area to avoid that the saliva from the parotid glands could influence the results. Thereafter, the minor salivary glands regions were carefully dried with compressed air, and subsequently, the examiners placed a pre-weighed Schirmer's test strip (Ophthalmos Industries, São Paulo, SP, Brazil). To avoid unwanted stimulation, strip positioning was gentle, with no finger or instrument pressure. The strip remained in place for one minute and then, upon removal, it was immediately weighted again to avoid weight loss from evaporation. The net weight was obtained calculating the difference between the second and the first weight.

**Figure 1:**
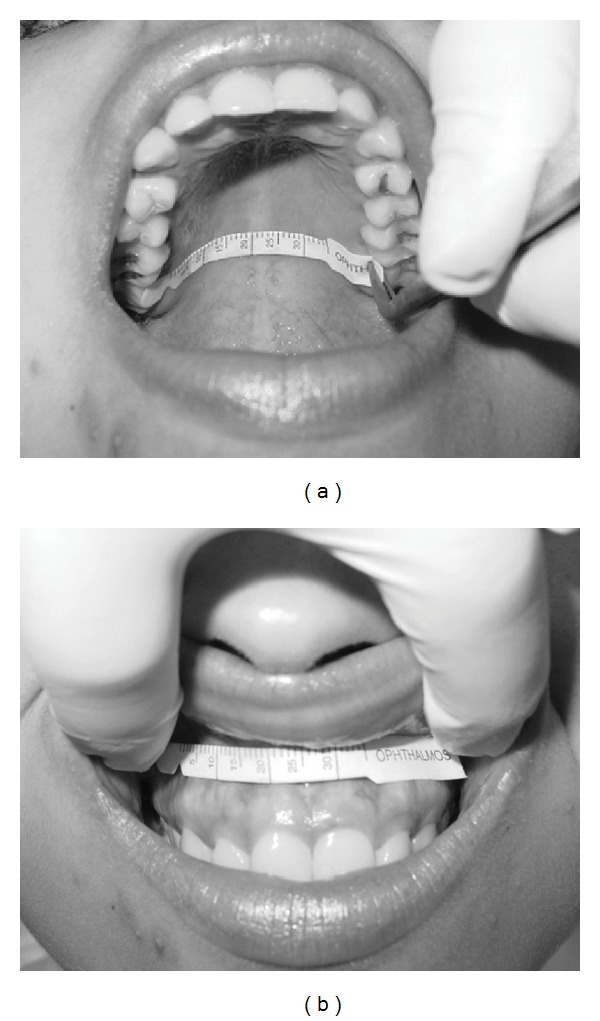
(a) Schirmer's strip paper inserted over posterior palatal region. (b) Insertion of the Schirmer's strip paper before placing the lip upon it to measure the saliva of the upper lip mucosa.

The saliva of the soft palate was first collected and subjects were instructed to keep the mouth open and to breathe through the nose during the collections to avoid that vapor from exhaled mouth air could affect the results. Afterwards, the saliva of the upper labial mucosa was collected. The collection area of PS was the soft palate between the left and the right second molars (Figure 1(a)), while for LS was the gingival mucosa between the apices of the left and right canines (Figure 1(b)), allowing contact with the overlying lip.

As the total area covered by the strip was 3 cm^2^, the value of the secretion rate was divided by 3 to get the salivary flow rate per cm^2^. However, the strip was previously cut in cases where the distance between the molars was less than the length of the paper strip, and a new calculation of its area was performed. The unit of measurement was expressed in microliter per square centimeter per minute (*μ*L/cm^2^/min).

### 2.3. Collection of Whole Saliva

UWS was collected by the draining method [12], while SWS was obtained by mechanical stimulus by chewing a piece of a sterilized cylindrical silicone (5 mm in diameter and 1 cm in length) tied to a dental floss. Both collections were performed during a five-minute period. The participants were instructed not to swallow the saliva during the collection. Nevertheless, they could spit the saliva into a previously weighed tube collector as many times as they needed during this period. The sample was weighed and the value was divided by the period of the collection to be expressed in milliliters per minute (mL/min).

### 2.4. Statistical Methods

Data analyses were performed with Statistical Package for Social Sciences (SPSS version 20.0 for Windows, SPSS Inc./IBM Group, Chicago, USA). All tests were two-sided and the level of 5% was required for statistical significance. The normality and homogeneity of variances of age and the types of saliva (UWS, SWS, LS and PS) were performed using the Kolmogorov-Smirnov and Levene tests, respectively. ANOVA was used to verify differences for those variables among groups of patients and Games-Howell posthoc test was performed because neither the population variances were assumed, nor the sample sizes were equal. Pearson's correlation test was used to verify the existence of relationship between types of saliva. Mean differences of types of saliva between xerostomia complainers with non-complainers were assessed with independent *t*-test. Differences in prevalence of individuals with and without xerostomia among groups were tested by chi-squared test.

## 3. Results

A total of 144 individuals were enrolled in the study: 119 female mean, age (±SD) 48 ± 16 years, and 25 male, mean age 46 ± 23 years.

The samples comprised of 24 subjects in the* severe hyposalivation group*, 78 individuals in the* mild hyposalivation group*, and 42 participants in the* normosalivation group*.

Moderate negative correlations between age and types of saliva were found, and the correlation with the UWS was the highest one (*r*_*s*_ = −0.464; *P* < 0.001), while the lowest correlation was with PS (*r*_*s*_ = −0.054; *P* = 0.523) (Table 1).* The mean age difference among and between groups was statistically significant* (Table 1)* and normosalivation group was comprised of subjects with lower ages* (38.8; ±13.8)* than other groups* (Table 2). Positive correlations occurred between all types of salivary flow rates. Moderate correlation occurred between UWS with SWS (*r*_*s*_ = 0.717; *P* < 0.001) and UWS with LS (*r*_*s*_ = 0.470; *P* < 0.001) (Table 1).

The mean value and the 95% confidence interval of PS flow rate for each group were 1.01 (0.68 to 1.34), 1.72 (1.31 to 2.13), and 2.44 (1.66 to 3.22), respectively (*P* = 0.014). The flow rates of LS were 3.20 (2.46 to 3.94), 5.86 (4.96 to 6.75), and 9.08 (7.63 to 10.53) (*P* < 0.001). Differences between groups were statistically significant for all types of salivary flow, except between* mild hyposalivation group* and* normosalivation group* for PS (*P* = 0.239) (Table 2).

The lowest means of salivary flow rates were found among xerostomia complainers that comprised all groups (Table 3). Prevalence of xerostomia complainers was higher in* severe hyposalivation group* when compared with the other groups (Table 4) (*P* < 0.001).

## 4. Discussion

The main objective of the study was to establish referential range values for the flow rate of the soft palatal saliva (PS) and upper labial mucosal saliva (LS) by the validation of a simple method of assessment, in a manner that it could be routinely used during clinical practice and be tested in SS patients in future study. Hence, firstly, it was checked whether correlation existed between UWS and other types of salivary flow rates, since the parameters used for the formation of the groups were based on UWS and SWS flow rates. Afterwards, the differences of salivary flow rates were assessed to verify whether LS and PS flow rates were statistically different to allow the establishment of referential range values.

Regarding the correlation of PS flow rate with UWS flow rate, a weaker correlation (*r*_*s*_ = 0.181) was found when comparing with another study that found higher correlation (*r*_*s*_ = 0.563) in women with normal salivary flow rate [26]. This might be explained by the difference of sample sizes, which was smaller (*n* = 30) than the one of our study, and probably because they enrolled only subjects without complaints suggestive of salivary gland dysfunction. The positive correlation between UWS with SWS flow rates (*r*_*s*_ = 0.717) found in this study is corroborated by a previous investigation that enrolled 78 subjects and used the same kind of stimulus we used to collect SWS (mechanical stimulation) [27]. This strong positive correlation can explain some clinical findings of dry mouth, like atrophy of tongue papillae, rampant dental caries, candidiasis, and other findings, in cases where only UWS or SWS is assessed and low amount of saliva is found.

Nevertheless, there is still a need to elucidate the reason of xerostomia complaint when sialometry of whole saliva shows normal flow rates. In this context, the modified Schirmer's test was capable of detecting different mean values for both LS and PS flow rates among groups (Table 2) and between subjects with and without xerostomia complaint (Table 3).

As previously demonstrated by other studies, our results showed that palatal saliva output presented lower rates when compared to upper labial output (Table 5) [17, 23]. Although further investigations are still required, the values obtained in this study may be of some help in establishing referential values as cut-off point. It means that values (*μ*L/cm^2^/min) below 3.94 for LS flow rate and 1.34 for PS flow rate can be indicatives of severe hyposalivation, while values above 7.63 for LS flow rate and 1.66 for PS flow rate can signalize normosalivation. Regarding the referential values for mild hyposalivation, the 95% CI of LS flow rate presented different values ranges from those found in hyposalivation and normosalivation groups, while the PS flow rate did not. Furthermore, the existence of a stronger correlation between UWS flow rate with LS than with PS flow rate found in our results makes it reasonable to assume that LS flow rate may be considered more reliable to predict hyposalivation than PS flow rate.

The methodology proposed in this study can be used as an additional tool to diagnose the early functional decline of salivary glands, which occurs during the intake of some medicines, radiotherapy, and in some systemic diseases like diabetes, SS, and others. A study of more than 600 patients suspected of having SS has found that 15% of those with primary SS and 26% of those with secondary SS did not present xerostomia complaint [28]. It is important to emphasize that the biopsy of minor salivary glands has been referred to as valuable tool for SS diagnosis [29]. However, some patients may not show significant lymphocytic infiltration, while others, without that disease, may present focal sialadenitis [29–31]. A study conducted in 10 patients with primary SS with their matched healthy controls revealed a reduction of labial saliva flow in almost 40% in the Sjögren's group [32]. In this context, it could be postulated that decreased secretion of minor salivary glands can be considered a risk indicator that may represent the first sign of some diseases that have had not been previously suspected. Nevertheless, further studies are still necessary.

It is known that minor glands flow rates are unaffected by a single gustatory stimulant [20]. On the other hand, the number of consistent information regarding reference values of minor glands flow rates is still limited. An important issue to be considered in future investigations is the establishment of references values ranges for both UWS and SWS flow rates, in order to consider a subject as hypo-or normosalivator before comprising groups of analysis when the aim of the study is to verify minor salivary glands' flow rates. We found only three studies that defined the stratification of groups according to their UWS and SWS to verify the secretion rates of minor glands [22, 23, 32], one [23] of them took into consideration the presence of xerostomia complaint. We also observed that other studies did not collect UWS and/or SWS [14, 17, 21], and nor explained how xerostomia complaint was assessed, though they stated that no participant reported complaints are suggestive of salivary gland dysfunction [17]. Moreover, some studies that used Periotron, which is reliable equipment, could not provide comparable information about minor salivary glands flow rates among them [17, 22, 23, 26, 32].

Another imperative issue that should also be highlighted, besides the establishment of range values for hypo-and normosalivation for minor glands flow rates, regards the mucosa areas to be assayed. The standardization of anatomical sites is extremely important to allow future comparisons due to the ample variation among protocols designs (Table 5). The mucosal areas chosen for our study were based on previous researches that suggest that the labial and palatal gland saliva might affect subjective feelings of dry mouth both in individuals with normal and subnormal whole saliva flow [22, 23].

There is a study that found the mean (SD) secretion rate of PS in normosalivators of 0.68 (±0.32) and states that this value is similar with or without stimulus [20]. This flow rate value is lower than the one found in our study 2.44 (±2.51). Nevertheless, besides the difference of the used methodology, it should be considered that the mean value of UWS flow rate of that study is also lower (0.54 ± 0.19) than the one found in this study (0.73 ± 0.35) and the sample size (*n* = 14) they have studied was smaller than ours (*n* = 42). Furthermore, although the collections were performed in the morning, they did not mention the time of the morning (Table 5).

A study [22] achieved with Periotron to access minor glands saliva flow rate, and conducted in stratified groups of UWS flow rates showed different results from ours. They did not collect SWS but were considered as* severe hyposalivation group* subjects that could not secrete saliva in the unstimulated state, while our study took into consideration both USW and SWS values to classify subjects into the severe hyposalivation group. Furthermore, their* mild hyposalivation group* was comprised of subjects with UWS flow rate of <0.15 mL/min, while our study considered those subjects with values ≥0.1 but <0.3 mL/min. Paradoxically, despite that study considers lower values than this one for UWS, the mean values in the* severe and mild hyposalivation groups* for PS flow rate (2.5 ± 3.1 and 2.8 ± 2.6, resp.) were higher than those found in our results (1.01 ± 0.79 and 1.72 ± 1.82). A possible explanation for this could be the pressure they used to fix the filter paper strip during saliva collection, which might have caused an extra stimulus upon minor salivary glands or the absence of cotton rolls on Stensen's duct area to avoid influence of saliva from the parotid glands.

Regarding age, our results showed statistically significant negative correlations among types of salivary flow rates, with the highest one being with UWS followed by LS. Even though the difference in mean age among and between groups was significant, comprising subjects with higher ages in group I than in other groups, it is not possible to state that age solely can explain the occurrence of hyposalivation or xerostomia. It is well established in the literature that these problems usually increase with age. The usage of several medications may alter salivary patterns and although polypharmacy may occur in all age groups, it is a common occurrence in elderly people [33]. As previously demonstrated, lower rates of saliva secretion are often present in xerostomia complainers [9] and our results were in accordance with this finding. The prevalence of xerostomia complainers was higher in severe* hyposalivation group* (88%), however it was also observed on subjects with* mild hyposalivation* (44%) and* normosalivation* (24%).

The literature shows that a decrease of saliva, mainly from the posterior palatal and the labial mucosa regions, may exacerbate the discomfort of dry mouth sensation even in the presence of normal major salivary gland function [23, 34]. It has been suggested that the cut-off value of palatal saliva for the occurrence of xerostomia is below 3 *μ*L/cm^2^/min [35].

Nevertheless, our results have shown that the 95% CI of PS flow rate found in xerostomia complainers and non-complainers encompassed a similar range of values (Table 3). However, the mean and the 95% CI were significantly lower in xerostomia complainers for LS flow rate (5.17; 4.06 to 6.23) when compared to non-complainers, which reinforces the usefulness of LS flow rates as an additional diagnostic tool.

We also performed (data not shown) an attempt to find an explanation for the 10 cases of xerostomia complaints in* normosalivation group* and also for the absence of it in those 3 subjects from* severe hyposalivation group* (Table 4). From those 10 cases of xerostomia in the* normosalivation group*, 5 (50%) presented their PS flow rate values that ranged from 0.07 to 1.0 (data not shown), which is considered as severe hyposalivation according to our findings. Additionally, another 2 (20%) subjects presented LS flow rate values of 4.7 and 5.93, respectively (data not shown), which were considered as mild hyposalivation in our results. Thus, 7 (70%) of those xerostomia complainers in the* normosalivation group* presented low salivary flow rates of LS or PS. Regarding those 3 subjects from the* severe hyposalivation group* that did not complain of xerostomia, it was verified that although a female participant had no UWS and only 0.1 mL/min of SWS, her LS flow rate (5.8 *μ*L/cm^2^/min) (data not shown) was compatible with normosalivation in accordance with our findings. Concerning the other two subjects, the second one presented SWS flow rate of 0.64 mL/min. These findings could be the possible reason for the absence of xerostomia in those two subjects. However, the third one had low flow rates for all types of saliva that were collected in this study. Nevertheless, saliva from other mucosal areas was not assessed and can possibly be the explanation for such paradoxes found in some cases from* severe and normosalivation groups*.

It should be highlighted that some studies use visual analogue scale to evaluate the efficacy of some therapies for salivary glands stimulation, and it is not uncommon the report of xerostomia improvement even without the augment of UWS and SWS flow rates. Therefore, the proposed method might explain this “paradox” by evaluating the LS and PS flow rates in future studies.

Filter paper strips have already been validated to blot saliva secretions [17, 19, 36–38] and the modified Schirmer's test has been used to measure resting whole saliva as also to distinguish subjects with and without xerostomia [19]. Furthermore, the strip is not expensive and has wide patient acceptability [19]. Nevertheless, for the evaluation of minor salivary glands flow rates, the practitioner needs to have a very accurate balance to weigh the strip before and immediately after saliva collection. A very favorable aspect of this methodology is that this type of equipment has been used in a variety of health activities and it is easy to be acquired. Moreover, its usage does not require specific training, and technical maintenance is rarely needed. These are positive aspects for the applicability of the proposed technique.

It has been reported that salivary gland dysfunction can occur long before the development of xerostomia [39], and also that on the early stage of SS whole saliva flow rate may not show any loss [40]. It is interesting to mention that nine participants who met the criteria for SS based on the European criteria presented both LS and PS flow rates below normal reference values (data not shown).

In this sense, health care professionals should not underestimate salivary evaluation by restricting the exam only to UWS and SWS but should also evaluate the saliva from minor salivary glands. Additional comparative studies in healthy subjects and in SS patients using the proposed method with similar design are needed. Furthermore, longitudinal studies should be also performed in patients with suspect SS to support the relevance of this test for the early diagnosis of SS. Therefore, this methodology for monitoring the salivary flow rate of labial salivary glands may be crucial to diagnose, predict, and/or prevent some oral diseases and consequently, some systemic complications.

In conclusion, the test was valid to be used in the upper labial mucosa area to distinguish normosalivation from severe and mild hyposalivation and also to elucidate the range values of salivary flow rates for xerostomia.

## Figures and Tables

**Table 1:** Correlations among salivary flow rates.

		Age	UWS	SWS	LS	PS
Age	Pearson correlation	1	−0.464**	−0.367**	−0.453**	−0.054
	Sig. (2-tailed)		<0.001	<0.001	<0.001	0.523

UWS	Pearson correlation		1	0.717**	0.470**	0.181*
	Sig. (2-tailed)			<0.001	<0.001	0.030

SWS	Pearson correlation			1	0.373**	0.177*
	Sig. (2-tailed)				<0.001	0.034

LS	Pearson correlation				1	0.309**
	Sig. (2-tailed)					<0.001

PS	Pearson correlation					1
	Sig. (2-tailed)					

**Correlation is significant at the 0.01 level (2-tailed).

*Correlation is significant at the 0.05 level (2-tailed).

**Table 2:** Age and secretion rates of major and minor salivary glands among groups.

	SFR				*P* value	
	Severe hypo (*n* = 24)	Mild hypo (*n* = 78)	Normo (*n* = 42)	Total (*n* = 144)	Among groups*	Between groups**
Age^a^						
Mean	58.8	47.7	38.8	47.9	<0.001	0.001^†^; <0.001^‡^; 0.014^§^
SD	11.3	18.4	13.8	17.1		
95% CI for mean						
Lower bound	55.0	43.1	34.4	45.0		
Upper bound	62.5	52.2	43.0	50.7		
UWS^b^						
Mean	0.02	0.28	0.73	0.37	<0.001	<0.001^†,‡,§^
SD	0.03	0.14	0.35	0.33		
95% CI for mean						
Lower bound	0.01	0.25	0.62	0.31		
Upper bound	0.03	0.31	0.84	0.42		
SWS^b^						
Mean	0.21	0.71	1.62	0.89	<0.001	<0.001^†,‡,§^
SD	0.21	0.36	0.55	0.65		
95% CI for mean						
Lower bound	0.12	0.62	1.45	0.78		
Upper bound	0.30	0.79	1.79	1.00		
LS^c^						
Mean	3.20	5.86	9.08	6.35	<0.001	<0.001^†, ‡^; 0.001^§^
SD	1.76	3.98	4.65	4.38		
95% CI for mean						
Lower bound	2.46	4.96	7.63	5.63		
Upper bound	3.94	6.75	10.53	7.08		
PS^c^						
Mean	1.01	1.72	2.44	1.81	0.014	0.02^†^; 0.003^‡^; 0.239^§^
SD	0.79	1.82	2.51	1.98		
95% CI for mean						
Lower bound	0.68	1.31	1.66	1.49		
Upper bound	1.34	2.13	3.22	2.14		

Grouping variable: salivary flow rates (SFR).

^a^Years.

^b^mL/min.

^c^ *μ*L/cm^2^/min.

*ANOVA with 0.05 level of significance.

**Games-Howell post hoc test with 0.05 level of significance.

^†^Between groups I and II; ^‡^between groups I and III; ^§^between groups II and III.

**Table 3:** Salivary flow rates between subjects with and without xerostomia complaint.

	Xerostomia	Mean	Std. deviation	95% CI		*P* value*
				Lower bound	Upper bound	
UWS^a^	Yes	0.25	0.27	0.18	0.31	<0.001
	No	0.47	0.34	0.39	0.54	
SWS^a^	Yes	0.63	0.57	0.49	0.77	<0.001
	No	1.10	0.63	0.96	1.24	
LS^b^	Yes	5.17	1.45	4.06	6.23	0.003
	No	7.33	1.39	6.4	8.27	
PS^b^	Yes	1.4	0.59	0.97	1.84	0.023
	No	2.13	0.7	1.68	2.61	

^a^mL/min.

^b^ *μ*L/cm^2^/min.

*Independent *t*-test with 0.05 level of significance.

**Table 4:** Prevalence of xerostomia complainers.

		SFR				*P* value	Linear-by-linear association
		Severe hypo *n* (%)	Mild hypo *n* (%)	Normo *n* (%)	Total *n* (%)		
Xerostomia	Yes	21 (88)	34 (44)	10 (24)	65 (45)	<0.001*	0.000
	No	3 (12)	44 (56)	32 (76)	79 (55)		

*Chi-squared test *P* < 0.001 (Asymp. Sig. 2-sided).

Salivary flow rates (SFR).

**Table 5:** Comparison between the present trial and former studies of salivary flow of minor salivary glands under no stimulation.

	Study											
	Shern et al.* 1990 [20]	Sivarajasingam and Drummond^†^ 1995 [17]	Eliasson^†^ et al. 1996 [21]	Lee et al. 2002 [22]			Eliasson et al.^‡^ 2009 [23]			This study		
Method	Periotron	Periotron	Periotron	Periotron			Periotron			Schirmer test strip		
Total of participants	14	99	127	51			142			144		

Groups	Normo			Normo	Mild hypo	Severe hypo	Normo	Mild hypo	Low whole secretion	Normo	Mild hypo	Severe hypo

UWS^a^	0.54 ± 0.19			0.43 ± 0.33	0.1 ± 0.04	0	≥0.2	<0.2	<0.2	0.73 ± 0.35	0.28 ± 0.14	0.02 ± 0.03
SWS^a^							>1.0	>1.0	<1.0	1.62 ± 0.55	0.71 ± 0.36	0.21 ± 0.21
Age (mean age)	37	17 to 81	22 to 89 (55)	18 to 74 (44)			18 to 82 (56)			18 to 88 (48)		
Smokers			Some				Some			Some		
Fasting for collection (hours)		1		2			2			2		
Time collection	Morning	2 to 5 p.m.	8 to 12 a.m.	8 to 11 a.m.			8 a.m. to 1 p.m.			8 to 10 p.m.		
Period of collection (seconds)	30	30	Different/site	30			Different/site			60		

Mucosa sites^b,§^				Normo	Mild hypo	Severe hypo	Normo	Mild hypo	Low whole secretion	Normo	Mild hypo	Severe hypo

PS	0.68 ± 0.32	0.56 ± 0.25	0.91 ± 0.08	3.2 ± 2.9	2.8 ± 2.6	2.5 ± 3.1	0.7 ± 0.26	0.76 ± 0.33	0.73 ± 0.2	2.44 ± 2.51	1.72 ± 1.82	1.01 ± 0.79
ULS		1.4 ± 0.73								9.08 ± 4.65	5.86 ± 3.98	3.2 ± 1.76
LLS	0.92 ± 0.48	2.19 ± 1.0	4.79 ± 0.31				2.36 ± 1.16	2.83 ± 1.23	2.5 ± 1.26			
BMS	2.59 ± 0.94	2.98 ± 1.0	16.1 ± 0.7				15.8 ± 4.56	14.3 ± 3.96	12.36 ± 3.06			
LMS		3.03 ± 1.49										

*The mean value from the two collections was used.

^†^Did not collect UWS to distinguish normosalivators from severe hyposalivators or hyposalivators to compare those values with the secretion values of minor salivary glands.

^‡^Mild hyposalivation was considered <0.2 for UWS and 1.0 mL/min for SWS; low whole saliva secretion as <0.2 for UWS and <1.0 mL/min for SWS.

^§^PS: palatal saliva; ULS: upper labial saliva; LLS: lower labial saliva; BMS: buccal mucosal saliva; LMS: lingual mucosal saliva.

^a^Values are expressed in milliliter per minute (mL/min).

^b^Values are expressed in microliter per square centimeter per minute (*μ*L/cm^2^/min); ± standard deviation.

## Clinical Perspectives

Health care professionals should not underestimate salivary evaluation by restricting the exam only to UWS and SWS but should also evaluate the saliva from minor salivary glands. Future studies comparing labial salivary flow rate between healthy subjects and patients with diseases or under the usage of medications that can affect salivary flow rate by the use of the proposed method are needed to elucidate the usefulness of our method in the context of early diagnosis of salivary gland hypofunction. Furthermore, longitudinal studies should be also performed in patients with suspect of Sjögren syndrome to support the relevance of this test. Therefore, this methodology for monitoring the salivary flow rate of upper labial salivary glands may be an additional tool to diagnose, predict, and/or prevent some oral diseases and consequently, some systemic complications.
